# Aggressive Bimodal Communication in Domestic Dogs, *Canis familiaris*

**DOI:** 10.1371/journal.pone.0142975

**Published:** 2015-11-16

**Authors:** Éloïse C. Déaux, Jennifer A. Clarke, Isabelle Charrier

**Affiliations:** 1 Department of Biological Sciences, Macquarie University, Sydney, New South Wales, Australia; 2 Université Paris Saclay, Université Paris-Sud, CNRS, UMR 9197, Institut des Neurosciences Paris-Saclay, Orsay, France; University of Lincoln, UNITED KINGDOM

## Abstract

Evidence of animal multimodal signalling is widespread and compelling. Dogs’ aggressive vocalisations (growls and barks) have been extensively studied, but without any consideration of the simultaneously produced visual displays. In this study we aimed to categorize dogs’ bimodal aggressive signals according to the redundant/non-redundant classification framework. We presented dogs with unimodal (audio or visual) or bimodal (audio-visual) stimuli and measured their gazing and motor behaviours. Responses did not qualitatively differ between the bimodal and two unimodal contexts, indicating that acoustic and visual signals provide redundant information. We could not further classify the signal as ‘equivalent’ or ‘enhancing’ as we found evidence for both subcategories. We discuss our findings in relation to the complex signal framework, and propose several hypotheses for this signal’s function.

## Introduction

The concept of complex signalling refers to the integration of multiple components into elaborated signals [1]. The class of multimodal signals denotes displays composed of two or more signals emitted in different modalities [1–3]. According to Partan and Marler [2], multimodal signals can be classified based on the information content of their unimodal components and on their effects on receivers’ behaviours. When separate components elicit distinctly different responses, they should convey different information and are termed ‘non-redundant’. Depending on the type of responses elicited when the components are presented in combination, their effects can be further classified as independent, emergent, modulatory or dominant (for a detailed explanation of the various categories and summary tables see [2, 3]). Evidence of non-redundant signals exists for invertebrate [4, 5], anuran [6] and primate [7] species. Alternatively, redundant multimodal signals present the same type of information in different sensory modalities, and are thought to serve as ‘back-ups’, allowing greater accuracy of elicited responses ('back-up' hypothesis, sensu [8]). For instance, as each signalling modality differs in its advantages and limitations (e.g. visual channel: fast communication mode but a line of sight is required; acoustic channel: long-distance communication but easily eavesdropped on [9]), simultaneously sending redundant information through multiple channels may improve the signal’s detectability despite environmental variability [1]. Individual signals of redundant displays can be said to be ‘equivalent’ or ‘enhancing’ if the intensity of the responses remains constant or increases when signals are combined, respectively [10, 11].

Multimodal signals have been widely studied in the contexts of mate choice and aposematic displays (for reviews see [12, 13]), but evidence of multimodal communication in intraspecific agonistic interactions is limited in comparison (but see [6, 14–16]). Considering the high survival costs associated with contests (e.g. energy expenditure, injury or death [17]), multimodal signals may be widespread in agonistic communication that precede contests, if it confers advantages over unimodal communication. The ‘back-up’ multimodal signals hypothesis seems particularly relevant as increased accuracy in assessment of, and response to signals displayed during agonistic interactions will ensure that escalation into a contest is limited to situations where the benefits outweigh the costs. Alternatively, multimodal signals may provide non-redundant information such as fighting ability or motivation [7]. Furthermore, research on multimodal communication has focused primarily on arthropod, anuran, fish and bird species (see studies reviewed in [1, 13, 18]), with few studies looking at mammal species (but see [7, 19]) and none yet conducted on a canid species.

While agonistic interactions includes all offensive behaviours (i.e. aggressive/threat signals and attack) and defensive behaviours (i.e. submissive signals and flight), in this study we focus on aggressive (also termed ‘threat’) signals (i.e. signals produced in agonistic contexts that predict escalation toward attack [20]). Dogs’, *C*. *familiaris*, aggressive vocal sequences include growl and bark vocalisations uttered in succession (hereafter referred to as the ‘acoustic signal’). Several studies have investigated the potential of bark and growl vocalisations to convey cues of signallers’ individual identity, body size and/or intention or motivation and their effect on inter- and intra-specific receivers (for review see [21]). For instance, the acoustic characteristics of growl vocalisations vary according to the signaller’s body size [22]. Additionally, dogs appear to perceive these size-related cues as they will be more likely to look at a size-matched dog model upon hearing growl vocalisations [23] and will show different responses according to the perceived size difference [24].

Additionally, offensive individuals will display a range of aggressive visual signals that include (but are not limited to) exhibiting a rigid, erect body posture; piloerection; bared teeth; staring at the opponent; and gaping at the opponent [25–27]. While there is an extensive body of empirical studies on aggressive vocal signals, considerably less attention has been given to the visual displays concurrently produced. Early work on canid behaviour described the ontogeny and development of behaviours and facial expressions during agonistic interactions [25, 28] and comparative studies have described similarities and differences in social behaviours (including agonistic) among canid species [27, 29–32]. Observational studies of dogs indicate that aggressive visual displays can also be performed in play contexts, although the behavioural sequences are unpredictable, interspersed with behaviours normally produced in other contexts, and metacommunication signals advertising ‘play’ are given prior to and during play sessions [33, 34]. However, there has been no empirical study of the function of visual signals, whether alone or in combination with acoustic signals, or when produced in agonistic and/or playful contexts. Given the advocated importance of studying signals produced in different modalities as a whole, rather than in isolation, and the compelling evidence that complex signalling is widespread [1, 2, 35], it seems overdue that canids’ audio-visual signals be studied in combination. Particularly, dogs make an ideal model system to begin investigating the function(s) of canids’ bimodal signals, as they are readily available and their long history of close association with humans make them suitable subjects for controlled manipulative experiments.

As a first step in investigating dogs’ bimodal signals we aimed to categorize the aggressive display according to Partan and Marler’s [2] classification. Thus, we tested the effect of the acoustic and visual signals on receivers, when presented either in isolation or in combination. Given the wide range of information that can be conveyed by the acoustic and visual channels, we hypothesized that 1) these signals would provide redundant information and 2) when combined, they would have an enhancement effect on receivers’ responses. To test these hypotheses, we used the cue-isolation protocol, where stimuli from a single or few models (e.g. [6, 19, 36, 37]) are presented either in isolation (unimodal presentation) or in combination (bimodal presentation), a standard procedure successfully used for many taxa [3, 38–40]. There is compelling evidence that dogs respond to audio playbacks as though perceiving the information content [23, 24, 41, 42]. Cross-modal studies also demonstrate dogs’ abilities to extract information from photos or models of dogs [23, 43]. Additionally, dogs respond similarly to commands and pointing gestures given by a human either when present or when a real-sized video of the human is projected [44, 45] and show responses demonstrating their ability to use intraspecific cues from videos alone [46]. Together, these studies indicate that dogs can extract information content from audio and video stimuli and provide support for the use of audio-visual playbacks as a means of investigating dogs’ intraspecific signals. As such, we used a playback experimental procedure testing dogs’ responses to the video of a real-sized dog showing aggressive visual signals (visual-only), to the playback of the dog’s acoustic signal (audio-only), and to the combination of both signals (audio-visual).

## Materials and Methods

### Stimuli preparation and presentation

To obtain playback stimuli, we audio- and video-recorded six dogs from All About Dog Training (Leppington, NSW, Australia). This centre provides various dog training services including schutzhund training for guard dogs. This type of training includes three parts: obedience, tracking and protection. In all cases the training, which uses positive reinforcement, focuses on enhancing behaviours that are naturally expressed in dogs. With regards to protection, the procedure involves using classical conditioning to elicit aggressive signals (e.g. standing erect while staring, teeth snapping, growling and barking) under certain conditions and is not known to alter these displays. As such, it provides an ethical situation to record dogs that reliably show natural aggressive signals while controlling for their distance to and orientation toward the video camera. Video recordings were made using a Sony Handycam HDR-PJ760 camera mounted on a tripod, 85cm above the floor with the white balance manually adjusted before the session. Audio recordings were performed using a Fostex FR-2LE recorder and a Sennheiser shotgun ME67 microphone. Audio files were recorded in stereo at 48 kHz sampling rate, 32-bits depth and saved as.wav files. Audio levels were manually adjusted at the start of each recording session to prevent clipping and were then kept constant. All recordings were performed on the same day, between 10:00–12:00, in a 3.5x3m empty room. Dogs were attached to a 1m steel lead, and the recording equipment was placed in front of the dog, 2.5m away. Each dog was recorded for up to 10min while the trainer evoked aggressive responses.

Of the six dogs recorded, three (a Rottweiler, a German shepherd and a Rottweiler x German shepherd cross) exhibited at least 30s of uninterrupted aggressive visual displays, with the matching acoustic signal (i.e. growl and bark vocalisations successively produced) not being degraded or masked by background noise and were thus selected as exemplar dogs. If more than 30s of continuous, good quality audio and video was available, we selected that section where the dog was most often looking toward the camera, such that the exemplar dog would appear to direct its behaviours at the test subject. For each exemplar dog, we isolated these 30s video clips and saved them as mp4 files. The matching audio clips were also isolated, normalized in amplitude (peak normalization, -1dB) and saved as.wav files. The video and audio clips formed the video-only and audio-only stimuli respectively (S1 Video). We created audio-visual stimuli by synchronizing the audio and video files and saving them as mp4 files (S1 Video). We also created an audio-visual control stimulus, which was a 30s video clip of the empty room, where the exemplar dogs were filmed, synchronized with 30s of background noise (i.e. bird calls and traffic noise) recorded outside of the shelter facility. All stimuli manipulations were performed using Audacity 1.3.13 (http://audacity.sourceforge.net/) and VideoPad 3.34 (NCH software).

To present stimuli using a consistent procedure, we embedded audio, visual and audio-visual clips in PowerPoint (Microsoft Inc.) presentations. All presentations were composed of two slides with a black background. The first slide was empty (i.e. black slide with no sound), while the second slide contained the embedded stimulus (i.e. the control, audio, video or audio-visual clip). An additional PowerPoint presentation composed of two empty black slides was used during the habituation phase of the experiment (see *Playback procedure* section).

### Subjects and experimental design

We used shelter dogs because they are readily available for testing, and a recent study demonstrated that there was no evidence for an effect of past history or current shelter environment on dogs’ responses to conspecifics’ aggressive acoustic signals [47]. These dogs were kept at the Animal Welfare League Kemps Creek shelter, Kemps Creek, NSW, Australia, during July 2014—May 2015, and individually housed in kennels with both indoor and outdoor areas. They were locked inside overnight, but stayed outside throughout the day and were walked four times a day. Dogs were fed dry dog food or a mixture of dry/wet dog food daily and water was provided ad libitum. A total of 54 neutered dogs of various breeds were used in this study, including 27 dogs of each sex. All dogs had been in the shelter for less than one year and more than two weeks, and had had previous experiences with other dogs (such as originating from multi-dog households before being surrendered and/or having regular interactions with other dogs at the shelter). Their reactivity toward conspecifics was also assessed by a shelter professional through staging an encounter with another dog and recording whether they displayed appropriate greeting behaviours (i.e. sniffing each other’s body parts, often accompanied by tail wagging) and no high levels of agonistic behaviours (e.g. crouching of the body, tail tucked under the belly, teeth showing or raised hackles, production of bark or growl vocalisations). Only those dogs that had passed the test were included in the experiment. Dogs’ characteristics (sex, age and weight) were obtained from the shelter’s records. Because body size is often an important factor for contest outcome, and there is evidence that dogs can adapt their responses when the opponent is perceived to be bigger or smaller [24], it was important to keep the signaller: receiver size difference consistent. Thus, all dogs included in the experiment were smaller than the exemplar dogs. While this procedure did not remove the potential impact of size assessment on dogs’ responses, it ensured that the assessment would be kept consistent across all test subjects and treatments.

Each of 45 dogs were randomly assigned to one of the three treatments (audio-only, video-only or audio-visual) and to one of three exemplar dogs, such that we had a full factorial design with five test dogs in each combination of treatment by exemplar dog level. Because our experiment involved introducing dogs to a dark room with only the presence of an unfamiliar person, this set-up could be stressful in itself, and induce dogs to react to a suddenly appearing audio and/or visual stimulus. Thus, another randomly selected nine dogs were presented with an audio-visual control stimulus in order to test that the experimental procedure did not impact dogs’ behaviours. While dog assignment was random, groups were counter-balanced with regards to sex, age and body size. All animals were subjected to an initial habituation trial. This procedure ensured that any potential impact of the testing environment was reduced and allowed us to monitor dogs’ behaviours to further confirm their inclusion in the experiment.

### Test room

All tests were conducted on the shelter grounds between 09:00–16:00, in an 8x3m testing room. A projector screen was placed on the floor against one wall. A playback speaker (Cube Street, Roland Corp.) was set behind the screen, 50cm above the floor. A video projector (Epson WXGA EB-1760W) was placed 2.8m from the screen, 1.4m above the floor and facing the middle of the projector screen. The experimenter sat on a chair next to the table supporting the video projector and operated the connected laptop (HDMI connection). To ensure that the video projections were clearly visible, we turned off all the lights and the room’s only window was covered with blinds.

To record behavioural responses, we used three cameras ensuring that the room’s entire area was in view. These video-only security cameras (infra-red lights, full HD, wide angle; Swann Co.) were connected to a network video recorder (Swann NVR4-7200, 4 channel 1080P) and a monitor, which were located in an adjacent room. An additional sound-enabled Sony Handycam HDR-CX130 camera was used to record dogs’ vocal responses. The testing room also included a fan for ventilation between trials, and water was provided ad libitum.

### Playback procedure

Each dog was subjected to one habituation trial and one test trial on consecutive days, at the same time of day. Tests were conducted between July 2014 and May 2015, with each trial lasting 5min. On the test day, video projections were adjusted to present life-sized dogs, such that the Rottweiler exemplar dog was set to measure 70cm at the shoulder. Audio levels were adjusted to 70-75dB SPL (range: 65-78dB SPL across all stimuli), measured at 1m using a RadioShack 33–2055 digital sound level meter (peak amplitude, C weighting on slow setting).

At the start of a trial, the video projector showed the first (black) slide of the PowerPoint presentation and the experimenter sat on the chair. A dog was introduced into the room and allowed to explore the surroundings for the first 2min. Throughout the trials, dogs were off-leash, such that we did not restrict their ability to move about, and the experimenter was the only person present in the room. The subject was then showed a treat (puppy biscuits, IAMS Company) to encourage it to approach the experimenter. This procedure controlled for the subject’s attention immediately prior to stimulus presentation. It also ensured that all subjects were within 1m of the screen at the time of presentation, such that all dogs had the means to detect the playback stimuli. The treat was then placed in front of the screen and, while the dog ate the treat, we presented the second slide of the PowerPoint presentation; which showed a black, silent slide during habituation trials but contained an embedded stimulus (control, audio-only, video-only or audio-visual) during test trials. After the stimulus ended, the dog was kept in the room for an additional 2.5min. To avoid providing behavioural cues to the dogs, the experimenter sat facing away from the screen with eyes closed and both arms and legs crossed throughout the trial.

### Behavioural measures

Dogs’ responses were scored from the videos recorded by the three security cameras. Video files were viewed at ¼ of the normal speed, using the software provided with the video recorder (SView Player 6.0.0.4), as it allowed displaying all three videos simultaneously. For each dog, we scored the start and end time of all the behaviours displayed pre-stimulus presentation and during stimulus presentation with each period lasting 30s (see S1 Table, for behaviours scored and their descriptions).

Dogs consistently exhibited four of the 13 behaviours scored (i.e. ‘explore’, ‘approach’, ‘retreat’ and ‘gaze’, see below for descriptions and S1 Table). A second observer, who was naive to the research question and to the experimental design, independently scored 40% of the videos, with respect to these four behaviours. Pearson correlations were used to investigate agreement levels between observers’ ratings. Correlation coefficients averaged 91.5% (range: 86–96%) across all four behaviours, demonstrating high levels of inter-observer agreement and thus confirming the reliability of our scoring method. From these four behaviours we created five behavioural responses, which were used for subsequent quantitative analyses: 1) proportion of exploration, 2) total response proportion, 3) proportion of gazing behaviour, 4) proportion of motor behaviours and 5) direction of the motor response.

#### Proportion of exploration

When a dog was moving freely around the room, in a relaxed posture (i.e. flat back as opposed to crouched/hunched, legs straight and fluid walking/trotting locomotion–without focusing on ear/tail posture as this can be influenced by breed-specific morphologies), with the head often held low to the ground and/or sniffing the ground, walls or objects, we categorized this behaviour as ‘explore’. The proportion of exploration was calculated as the time (s) spent exploring over total period duration (s).

#### Total, gazing and motor response proportions

We defined ‘gaze’ as the dog standing immobile in an alert posture and maintaining its head and eyes directed at the screen while the stimulus was being presented. A dog was scored as ‘approaching’, when upon stimulus presentation and while looking at the screen, it took two or more steps toward the screen. A ‘retreat’ score was given when after approaching and/or gazing at the screen, the dog took two or more steps away (while facing the screen or not) from the screen. Both types of behaviour could be performed at a walk or faster pace. The total response proportion was the sum of the gazing, approach and retreat proportions.

Gazing and motor behaviours were mutually exclusive and times engaged in these behaviours varied among treatments. Thus, to allow for comparisons between conditions, we calculated the proportion of each behavioural response (i.e. gazing or motor response) while correcting for the proportion of the other response. The proportion of the gazing response was measured as: gazing (s) / (total period (s)–motor response (s)), while the proportion of motor behaviours was calculated as: (approach (s) + retreat (s)) / (total period (s)–gazing response (s)).

#### Direction of motor response

As dogs’ motor responses included both approach and retreat behaviours, we aimed to not only quantify the ‘total amount’ (i.e. proportion of motor behaviour) but also whether individuals were more likely to engage in ‘approach’ or ‘retreat’ behaviours depending on treatment type. As such, we calculated the difference between the proportions of ‘approach’ and ‘retreat’ behaviours. A negative score thus indicated that the dog had predominantly responded by retreating away from the stimulus, while a positive score was indicative of a dog predominantly exhibiting approach behaviours. A score close to zero reflected that either the individual spent a similar amount of time alternating between approach and retreat behaviours or that a dog had not shown a motor response.

### Statistical analyses

To test for differences among groups prior to stimulus presentation, we analysed the proportion of exploration displayed in the pre-stimulus presentation period, using a one-way ANOVA. We also aimed to investigate if the playback procedure affected dogs’ behaviours. Thus for each behaviour scored, we compared the proportion of dogs displaying the behaviour between the treatment (n = 45) and the control groups (n = 9), using Fisher’s exact tests. Additionally, to investigate if dogs’ responses were influenced by the presence of the experimenter we tested whether the likelihood of dogs looking at, or approaching the experimenter during stimulus presentation differed between treatments (chi-square test) and/or was significantly different from random chance (binomial tests).

To determine if the uni- and bi-modal stimulus elicited the same type of responses, we used Fisher’s exact tests to test for differences among treatments in the proportions of dogs that exhibited each of the 13 behaviours scored during stimulus presentation. To investigate the effects of the treatments on the intensity of the dogs’ responses we tested for differences in total response proportion, gazing response proportion, motor response proportion and in the direction of the motor response exhibited during the stimulus presentation period. As we had a full factorial design, we used two-way factorial ANOVAs, with treatment (audio-only, visual-only and audio-visual) as the first factor, and exemplar dog as the second factor, thus avoiding pseudoreplication. Significant ANOVA tests were followed by posthoc Tukey’s HSD pairwise comparisons. All analyses were performed in R version 3.34 (http://www.R-project.org/).

### Ethics statement

This research was conducted in accordance with the Australian Code of Practice for the Care and Use of Animals for Scientific Purposes (NHMRC, 2013). All procedures were approved under Macquarie University Animal Ethics Committee protocol number 2013/036.

## Results

### Habituation trial, control stimulus and pre-stimulus presentation period

By the end of the habituation trial, all dogs were observed to move freely around the room, and none exhibited behaviours associated with high stress levels (e.g. lowered posture, increased frequency of vocalisation, snout licking, paw lifting [48]) and were thus included in the experiment. Furthermore, there was no difference in the proportion of exploration among groups prior to stimulus presentation (F_3, 50_ = 0.88, *P* = 0.45).

There were significant differences in the type of behaviours displayed in the control and treatment conditions (Table 1). The majority of dogs displayed gazing (95.6%), approach (77.8%) and/or retreat (88.9%) behaviours upon presentation of a uni-or bimodal treatment, while few did so when presented with the control stimulus (Fisher’s *P* < 0.001). Dogs were also more likely to investigate the screen and show ambivalent behaviours in the treatment conditions (Fisher’s *P* ≤ 0.05). Finally, 33% of the dogs in the treatment conditions continued exploring the room during stimulus presentation compared to 100% of the dogs in the control group (Fisher’s *P* < 0.001).

**Table 1:** Percentage of dogs displaying the behaviours scored in the control (n = 9) and treatment (n = 45) groups.

Behaviour	Control	Treatment*	Fisher's *P*
Gaze	55.6	95.6	<0.01
Approach	11.1	77.8	<0.001
Retreat	11.1	88.9	<0.001
Explore	100	33.3	<0.001
Screen investigate	0	37.8	0.04
Ambivalent movement	0	33.3	0.05
Door-oriented	0	26.7	0.18
Vocalize	11.1	13.3	1
Alert	0	26.7	0.18
Human-oriented	44.4	31.1	0.46
Scent-marking	0	0	1
Drinking	11.1	2.2	0.31
Sitting	22.2	8.9	0.26

* Pooled data for the audio-only, visual-only and audio-visual conditions.

Of the 45 dogs tested with either the audio-visual, audio-only or visual-only stimuli, seven did not look at, or approach the experimenter from the beginning of stimulus presentation and until the end of the trial. Of the remaining 38 dogs, the likelihood of seeking the experimenter before the end of the stimulus presentation was not significantly different from chance (binomial tests, *P* > 0.05, all treatments) or among treatments (chi-square test, χ = 1.96, df = 2, *P* = 0.37). Thus, dogs were reacting to the stimuli and did not pay attention to the experimenter while the stimulus was being presented, whether or not the experimenter unintentionally gave cues away. These results confirm that dogs’ responses to the uni- and bi-modal treatment stimuli cannot be attributed to initial differences among groups or the experimental set-up and were not influenced by the presence of the experimenter.

### Behavioural response types

**Fig 1:**
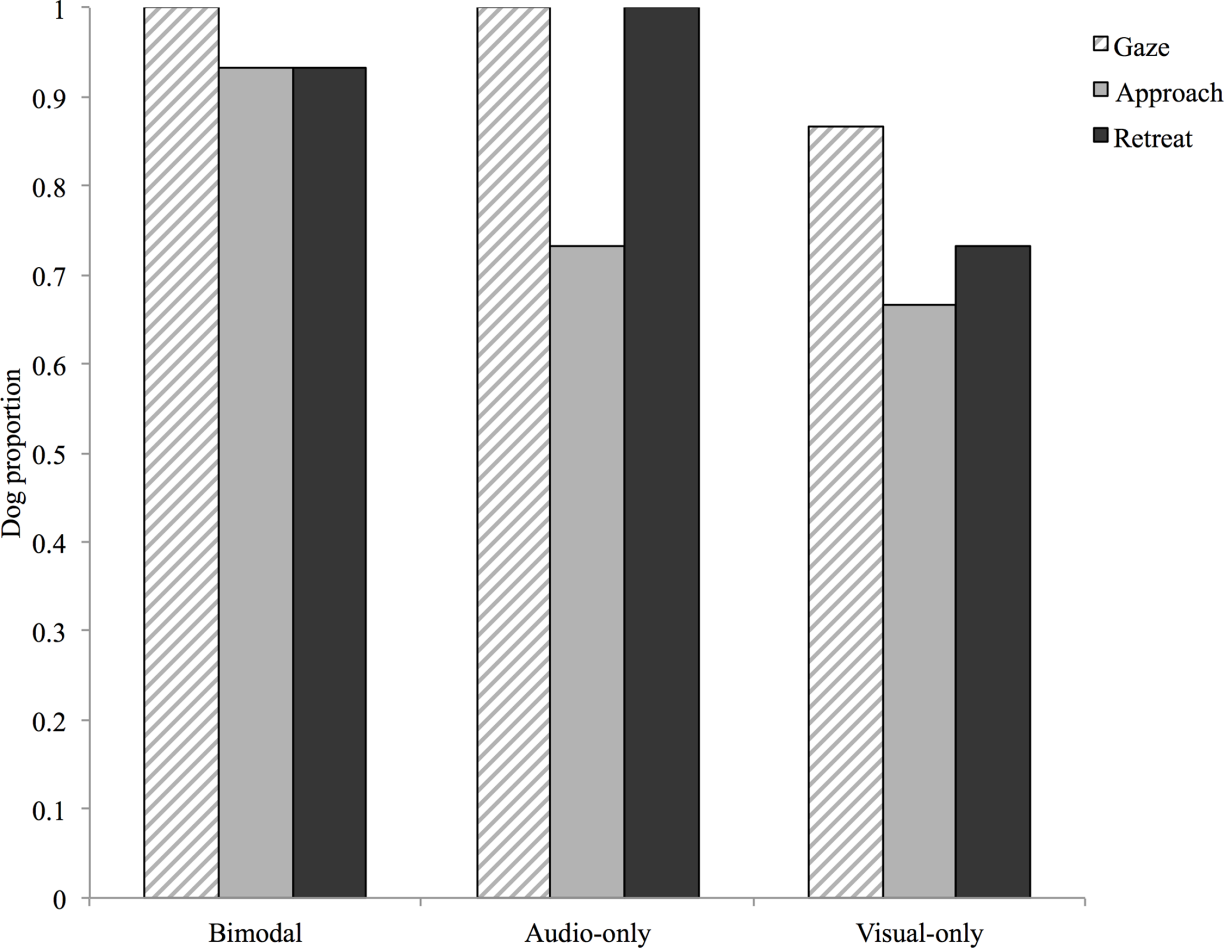
Dogs’ behavioural responses to aggressive audio and/or visual signals. Proportion of dogs (n = 15 per treatment) that exhibited gazing, approach and/or retreat behaviours in the bimodal (audio-visual), audio-only and visual-only treatments. Proportions did not significantly differ among treatments (Fisher’s *P* > 0.05, all three behaviours).

The proportion of dogs that exhibited a given behaviour was not different among treatments, for 12 of the 13 behaviours scored (Fisher’s *P* > 0.05). Dogs were more likely to display ambivalent behaviours in the audio-visual (53%) and audio-only (40%) treatments than in the visual-only group (7%, Fisher’s *P* = 0.02), although this behaviour was not reliably elicited. Conversely, behaviours consistently seen within treatments were equally elicited by all three stimulus types (gazing Fisher’s *P* = 0.32, approach Fisher’s *P* = 0.28 and retreat Fisher’s *P* = 0.11, Fig 1). Thus, dogs displayed qualitatively similar responses to uni- and bimodal stimuli.

### Total response proportion

**Fig 2:**
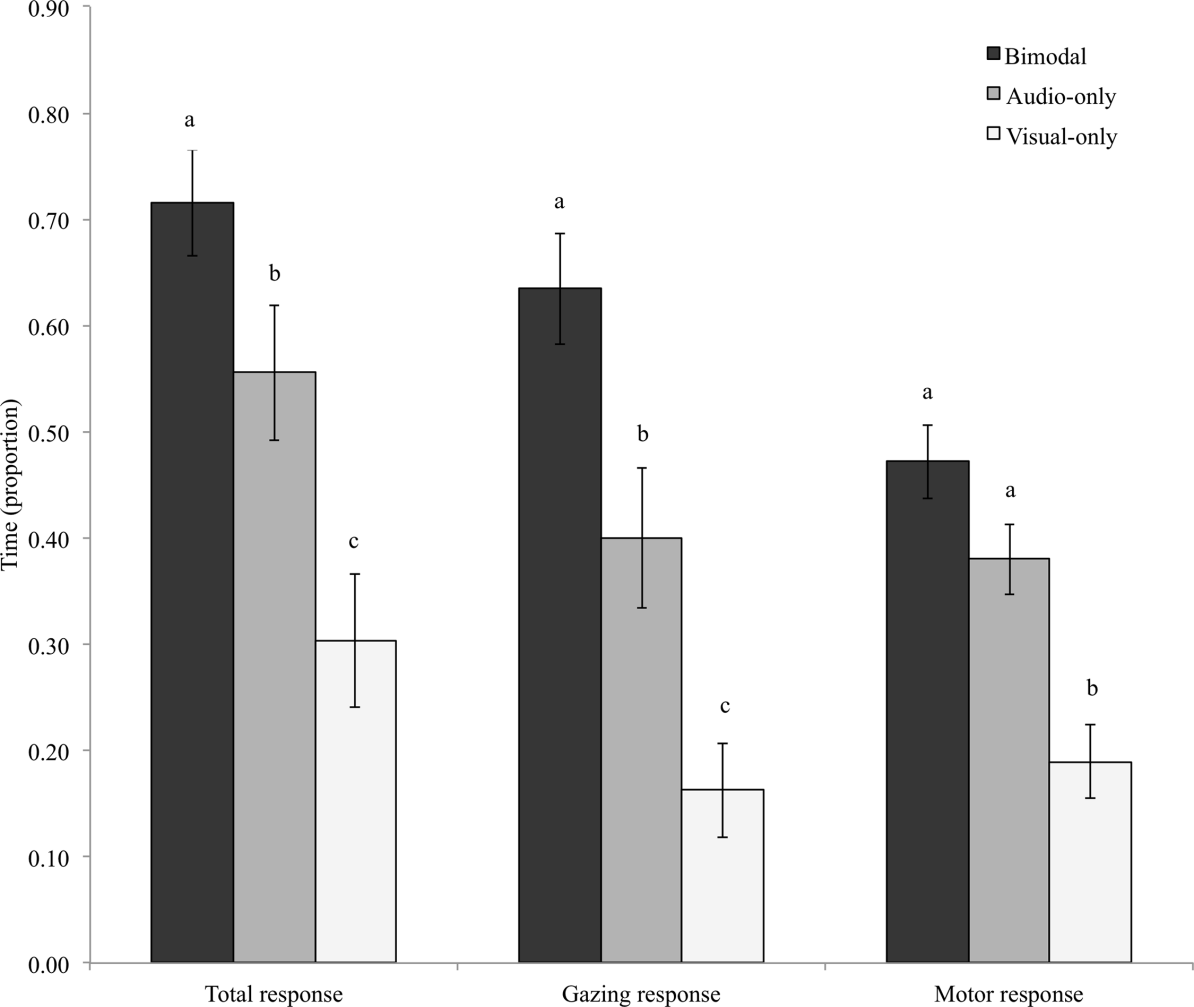
Intensity of dogs’ responses to aggressive audio and/or visual signals. Mean ± S.E. proportions for total, gazing and motor response proportions across treatments. Within response type, different letters indicate significant pairwise difference at Tukey’s HSD *P* < 0.05.

The proportion of total response (which included gazing, approach and retreat proportions) was significantly different among treatments (treatment: *F*_2, 36_ = 21.91, *P* < 0.001; exemplar dog: *F*_2, 36_ = 0.21, *P* = 0.81; treatment x exemplar dog interaction: *F*_4, 36_ = 2.37, *P* = 0.07, Fig 2). Dogs’ total response times were greatest in the bimodal condition, followed by the audio-only treatment, which also eliciting longer reaction times than the visual-only treatment (Tukey’s HSD *P* < 0.05, all pairwise comparisons).

### Proportion of gazing behaviour

The proportion of gazing behaviour was significantly different among treatments (treatment: *F*_2, 36_ = 19, *P* < 0.001) but not among exemplar dogs or for their interaction effect (exemplar dog: *F*_2, 36_ = 0.88 *P* = 0.43; treatment x exemplar dog interaction: *F*_4, 36_ = 1.82, *P* = 0.15). There was a greater proportion of gazing behaviour in the audio-visual treatment compared to both unimodal treatments (Tukey’s HSD *P* < 0.01). The audio-only treatment also elicited significantly more gazing behaviour than the video-only treatment (Tukey’s HSD *P* = 0.03, Fig 2).

### Proportion of motor behaviour and direction of motor response

The proportion of motor behaviours significantly differed among treatments (treatment: *F*_2, 36_ = 8.56, *P* < 0.001) but not among exemplar dogs (exemplar dog: *F*_2, 36_ = 0.09, *P* = 0.91) or their interaction (treatment x exemplar dog interaction: *F*_4, 36_ = 1.27, *P* = 0.3, Fig 2). The visual treatment significantly differed from both the bimodal and audio-only treatments (Tukey’s HSD *P* < 0.05), but they did not differ from one another (Tukey’s HSD *P* = 0.39). Thus, dogs spent more time approaching and/or retreating when the stimulus included at least the acoustic channel.

However, there was no difference in the direction of motor response among treatments (treatment: *F*_2, 36_ = 1.71, *P* = 0.2; exemplar dog: *F*_2, 36_ = 2.03, *P* = 0.15; treatment x exemplar dog interaction: *F*_4, 18_ = 0.79, *P* = 0.54). As such, the directionality of the response was not affected by the type or number of modalities presented.

## Discussion

We found that when presented with acoustic and/or visual aggressive signals, the majority of dogs tested displayed three primary behaviours: gazing at, approaching and/or retreating from the source, irrespective of the number of modalities used (S2 Video). Playbacks of growl vocalisations elicit similar behaviours and these responses correlate with changes in cortisol levels [47]. Thus, dogs in this study appeared to respond as though detecting the signals’ information content, irrespective of the modality used, and the results support our hypothesis that acoustic and visual signals are redundant. We also found that the total and gazing response times were greatest in the bimodal treatment followed by the audio-only and least in the visual-only treatment. Treatments that included the acoustic channel elicited the longest motor response but there was no difference among treatments with regards to the direction of the motor response. Thus our hypothesis that the bimodal signal would have an enhancement effect on dogs’ responses was partly supported (total response time and gazing response time), but we also found evidence of an equivalence effect (direction of the motor response) and a pattern of responses that cannot be classified as either (motor response proportion).

Redundancy in complex signals is a common and widespread phenomenon [10, 11, 19, 39]. In the case of dogs, the acoustic and visual signals may convey redundant information regarding individual identity, fighting ability (body size and/or weight) and/or fighting propensity; which are factors likely to affect contest outcome [17, 49, 50]. Indeed, dogs appear to discriminate among individually distinctive barks [42, 51]. They can also perform visual interspecific species discrimination, and generate cross-modal representations of humans [52, 53]. Thus they likely have the cognitive abilities required to discriminate among conspecifics using both visual and acoustic cues. Fighting ability can also be visually assessed from an opponent’s body size and dogs seem to perceive size-related cues encoded in growl vocalisations [22, 23, 43]. Additionally, the visual signals exhibited during agonistic interactions are highly ritualized in canid species [25], and could provide information on the interaction context and/or on the signaller’s motivation. Barks’ acoustic characteristics are also context-specific, potentially relating to signallers’ motivational and/or affective state, and dogs respond as though detecting such variations [42, 54].

Partan and Marler [2] argued that redundant multimodal signals could be further classified as either ‘equivalent’ or ‘enhancing’ based on whether receivers’ responses were quantitatively similar or amplified, respectively. However, we found that the effect of the bimodal stimulus was not consistent across all responses analysed, with evidence for enhancement (total and gazing response time), equivalence (motor response direction) as well as a pattern of motor response intensity not classifiable as either. As such, we cannot further categorize this signal according to their classification. Research on multimodal signals in other taxa also found patterns of responses that do not clearly fit within the redundant/non-redundant classification framework [10, 35]. This wide variation in multimodal signalling systems led Hebets and Papaj [1] to propose a research framework focusing on signals’ function(s). Indeed, a signal’s structure is adapted to its function and is the result of selective pressures impacting its information content (‘what’ is conveyed) and/or its efficacy (‘how’ is it conveyed). Selection may also favour inter-signal interactions (i.e. where the presence of one component influences the receiver’s response to another component [1]). Several of the functional hypotheses proposed by Hebets and Papaj [1], may explain the pattern of responses found in this study. These hypotheses are not mutually exclusive and it is likely that more than one type of selective pressure has impacted on the evolution of multimodal communication in dogs. In the following section we discuss these hypotheses with the aim to provide directions for future studies.

Redundant signals (whether enhancing or equivalent) are thought to function to increase receivers’ response accuracy [8]. This may be a result of environmental conditions that can affect signal transmission i.e. ‘efficacy backup’ or ‘efficacy trade-off’ [1], as is the case in the wolf spider *Schizocosa ocreata* [55]. Given that dog communication in agonistic contexts occurs over short distances (within a few metres), it seems unlikely however, that environmental variability would be an important force in driving the evolution of dogs’ bimodal aggressive signals. Alternatively, response accuracy may be improved through increased detectability, discriminability or memorability of signals by receivers [18]. Detectability refers to how easily signals can be separated from background stimuli by receivers. Signal detection can be studied by measuring receivers’ reaction times to stimuli, as faster signal detection will result in shorter reaction times [18]. The acoustic modality can promote signal detectability as it provides an instantaneous means of communication, and is less subjected to environmental blocking or constrained by receivers’ attention levels than the visual channel [9, 56]. Our experimental set-up was not designed to quantify dogs’ latency to react, but we noticed that it was highly variable in the visual-only condition (mean ± SD = 5 ± 5.58s), unlike in the audio-only (mean ± SD = 3 ± 1.19s) and audio-visual treatments (mean ± SD = 2.5 ± 1.3s). This trend suggests that, while the acoustic and visual channels convey redundant information, the acoustic modality may additionally function to increase the signal’s detectability, although formal investigation is required.

Furthermore, we found that there was a consistent pattern for the audio-only treatment to elicit longer response times than the visual-only treatment (Fig 2). Despite being redundant, acoustic signals are also more likely to elicit responses in female pigeons, *Columba livia*, than visual signals [39]. These variations in responses could be caused by differences in technologies impacting sensory stimulation (e.g. unnatural 2D video stimulus, [3, 39]) or by the unnatural experimental procedure, as in real situations it would be unlikely to see a dog behaving aggressively but not hear its vocalisations. Alternatively, receivers’ assessment of information could vary with sensory modality such that, despite being redundant, signals in a given modality may be of greater importance relative to signals in another sensory modality [57]. If this were the case, then we would expect that dogs presented with bimodal signals that violate the receivers’ expectations should display responses matching the information content of the signal that would dominate the association under natural conditions.

Another hypothesis of complex signal function, signal discriminability, proposes that multimodal displays function to increase a receiver’s ability to differentiate signals from a range of stimuli and respond accordingly [18]. Ultimately, the function of an aggressive signal is to provide receivers with the means to assess the quality (fighting ability and ‘motivation’) of the signaller before engaging in a contest. In the case of dogs, structurally similar acoustic and visual signals are produced across different contexts such as agonistic and play sessions. In these instances, the costs of discrimination errors (e.g. misclassifying an aggressive signal for a playful one) could be high, such that we would expect evolution to favour signals’ design that vary according to the context of production and thus allow for correct assessment of the interaction. To date, evidence of variations in growls’ acoustic structures according to context is conflicting [41, 58]. In contrast, during play sessions, dogs will perform the highly stereotypical ‘play bow’ posture, which is thought to serve as a solicitation signal and which is antithetical in form to the aggressive posture [34, 59]. Thus, as multimodal signals produced in antagonistic contexts share acoustic components that could imperfectly code for a signaller’s motivation (and/or the context of the interaction), visual components may function to increase signal discriminability.

Lastly, while in this study we considered the visual signal as a whole, it is formed of a suite of subcomponents (e.g. ears, tail and hackle posture, teeth and lips position/movement), which may have different relative importance in eliciting responses [15]. Future research investigating how the integration of these subcomponents affects receivers’ responses should provide further insights into the complexity and evolution of this canine bimodal display.

## Supporting Information

S1 File Summary statistics of the behavioural responses analysed. ANOVA tables including effect size measures (ETA and partial ETA squared) and summary statistics (mean ± SD) for each behavioural response analysed.
(PDF)

S1 Table Behaviours coded and their descriptions. (PDF)

S1 Video Examples of treatment stimuli: a) audio-only, b) visual-only and c) audio-visual. All stimuli have been trimmed to 10s.
(MP4)

S2 Video Example of a test dog’s responses. The subject was presented with an audio-visual stimulus.
(MP4)
