# Supplementary material for: Aggressive Bimodal Communication in Domestic Dogs, Canis familiaris
Source: PLoS One. 2015 Nov 16;10(11):e0142975. doi: 10.1371/journal.pone.0142975 (PMC4646621; doi:10.1371/journal.pone.0142975)
Supplement: S1 File — ANOVA tables including effect size measures (ETA and partial ETA squared) and summary statistics (mean ± SD) for each behavioural response analysed. (PDF) [file pone.0142975.s001.pdf]

ANOVA TABLES INCLUDING ETA AND PARTIAL ETA SQUARED

**TOTAL RESPONSE**	eta.sq	eta.sq.part	SS	df	MS	F	p
STI	0.49	0.55	1.30	2.00	0.65	21.91	0.00
EX_DOG	0.00	0.01	0.01	2.00	0.01	0.21	0.81
STI:EX_DOG	0.11	0.21	0.28	4.00	0.07	2.37	0.07
Residuals	0.40	NA	1.07	36.00	0.03	NA	NA

**GAZING RESPONSE**	eta.sq	eta.sq.part	SS	df	MS	F	p
STI	0.46	0.51	1.67	2.00	0.83	19.00	0.00
EX_DOG	0.02	0.05	0.08	2.00	0.04	0.88	0.43
STI:EX_DOG	0.09	0.17	0.32	4.00	0.08	1.82	0.15
Residuals	0.43	NA	1.58	36.00	0.04	NA	NA

**MOTOR RESPONSE**	eta.sq	eta.sq.part	SS	df	MS	F	p
STI	0.29	0.32	0.62	2.00	0.31	8.56	0.00
EX_DOG	0.00	0.01	0.01	2.00	0.00	0.09	0.91
STI:EX_DOG	0.09	0.12	0.18	4.00	0.05	1.27	0.30
Residuals	0.62	NA	1.30	36.00	0.04	NA	NA

**DIRECTION OF MOTOR RESPONSE**	eta.sq	eta.sq.part	SS	df	MS	F	p
STI	0.07	0.09	0.03	2.00	0.01	1.71	0.20
EX_DOG	0.09	0.10	0.03	2.00	0.02	2.03	0.15
STI:EX_DOG	0.07	0.08	0.03	4.00	0.01	0.79	0.54
Residuals	0.77	NA	0.30	36.00	0.01	NA	NA

TABLE SHOWING MEAN AND (SD) VALUES FOR EACH TREATMENT AND FOR EACH BEHAVIOURAL RESPONSE MEASURED

**BEHAVIOURAL RESPONSE**	Bimodal	Audio-only	Visual-only
Total response	0.72 (0.2)	0.55 (0.2)	0.3 (0.13)
Gazing response	0.63 (0.25)	0.4 (0.25)	0.16 (0.13)
Motor response	0.47 (0.24)	0.38 (0.17)	0.19 (0.13)
Direction of the motor response	-0.03 (0.09)	-0.05 (0.09)	0.01 (0.1)
