# Supplementary material for: Aggressive Bimodal Communication in Domestic Dogs, Canis familiaris
Source: PLoS One. 2015 Nov 16;10(11):e0142975. doi: 10.1371/journal.pone.0142975 (PMC4646621; doi:10.1371/journal.pone.0142975)
Supplement: S1 Table — (PDF) [file pone.0142975.s002.pdf]

S1 Table: description of all behaviours scored.

Behaviour	Description
Alert	The subject is standing still and watching its surroundings, without focusing on any specific feature.
Ambivalent movement	The subject is walking/running around the room using stereotypical behaviours such as back and forth movements, circling or parallel walking. The pace is faster than when exploring and the head is held above ground. No stopping to sniff the room is performed.
Approach	The subject takes two or more steps in a straight path, toward the screen.
Door-oriented	Door-oriented behaviours including watching the door, sniffing, scratching and/or jumping at the door.
Drinking	The subject drinks from the water bowl.
Exploring	The subject walks at a steady, relaxed pace around the room, the head is often low, close to the ground, with occasional stops to sniff the ground/object of interest.
Human-oriented	Behaviours performed toward the experimenter including approaching, sniffing, jumping on and/or licking the experimenter
Gaze	The subject looks at the screen in response to the stimulus, while standing still.
Retreat	The subject takes two or more steps in a straight path, away from the screen, may or may not be concurrently looking at the screen.
Scent-marking	The subject deposits either urine or faeces on the ground or objects present in the room.
Screen	The subject investigates the screen either through prolonged sniffing or by going behind it.
Vocalize	Barks, growls or whines.
Sitting	The subject sits either on its haunches or with its belly on the ground.
